# New Work on Natural History

**Published:** 1849-10

**Authors:** 


### NEW WORK ON NATURAL HISTORY.

Professor Troost, of the University of Nashville, who has been long engaged in the study of the geology of Tennessee is about putting to press a quarto volume on the Crinoids of that State. It is to be published in Boston, and will form a work of rare interest to the naturalist, and reflect credit upon the science of our country. Dr. Troost has been singularly successful in collecting the remains of this beautiful race of animals, and his forthcoming volume will be found to embrace a greater number of species than was known to the world before. His list comprises fourteen genera and about ninety species, nearly all of which have been found within the limits of Tennessee. The specimens from which the drawings for his work have been made are very perfect, which will enabie him to give a picture of the Crinoidea more complete and beautiful than any author has yet exhibited.

